# Biomineralization potential and biological properties of a new tantalum oxide (Ta_2_O_5_)–containing calcium silicate cement

**DOI:** 10.1007/s00784-021-04117-x

**Published:** 2021-08-12

**Authors:** F. J. Rodríguez-Lozano, A. Lozano, S. López-García, D. García-Bernal, J. L. Sanz, J. Guerrero-Gironés, C. Llena, L. Forner, M. Melo

**Affiliations:** 1 Hematopoietic Transplant and Cellular Therapy Unit, Instituto Murciano de Investigación Biosanitaria Virgen de La Arrixaca, IMIB-Arrixaca, University of Murcia, 30120 Murcia, Spain; 2 Department of Dermatology, Stomatology, Radiology and Physical Medicine, Morales Meseguer Hospital, Faculty of Medicine, University of Murcia, 30008 Murcia, Spain; 3 Department of Stomatology, Faculty of Medicine and Dentistry, Universitat de València, 46010 Valencia, Spain; 4 Special Care and Gerodontology Unit, School of Dentistry, IMIB Arrixaca, Campus Regional de Excelencia Internacional “Campus Mare Nostrum”, University of Murcia, 30008 Murcia, Spain

**Keywords:** Bioactivity, Ion-releasing materials, NeoMTA, Vital pulp therapy biomineralization

## Abstract

**Objective:**

The present study evaluated the biological effects and biomineralization potential of a new tantalum oxide (Ta_2_O_5_)–containing material designed for vital pulp therapy or perforation repair (NeoMTA 2), compared to NeoMTA Plus and Bio-C Repair.

**Material and methods:**

Human dental pulp stem cells (hDPSCs) were exposed to different eluates from NeoMTA Plus, NeoMTA 2, and Bio-C Repair. Ion release from each material was determined using inductively coupled plasma-optical emission spectrometry (ICP-MS). The biological experiments performed were MTT assays, apoptosis/necrosis assays, adhesion assays, migration assays, morphology evaluation, and reactive oxygen species (ROS) production analysis. Biomineralization was assessed by Alizarin red S staining. Finally, osteo/odontogenic gene expression was determined by real-time quantitative reverse-transcriptase polymerase chain reaction (RT-qPCR). Data were analyzed using one-way ANOVA followed by Tukey’s multiple comparison test.

**Results:**

NeoMTA 2 displayed a significantly higher calcium release compared to the other materials (*p* < 0.05). When hDPSCs were cultured in presence of the different material eluates, all groups exhibited similar hDPSC viability and migration rates when compared to untreated cells. Substantial cell attachment and spreading were observed in all materials’ surfaces, without significant differences. hDPSCs treated with NeoMTA 2 displayed an upregulation of ALP, Col1A1, RUNX2 (*p* < 0.001), ON, and DSPP genes (*p* < 0.05), and showed the highest mineralization potential compared to other groups (*p* < 0.001). Finally, the more concentrated eluates from these materials, specially NeoMTA Plus and NeoMTA 2, promoted higher ROS production in hDPSCs compared to Bio-C Repair and control cells (*p* < 0.001), although these ROS levels did not result in increased cell death.

**Conclusions:**

The new tantalum oxide (Ta_2_O_5_)–containing material shows an adequate cytocompatibility and the ability to promote biomineralization without using chemical osteogenic inducers, showing great potential as a new material for vital pulp therapy.

**Clinical relevance:**

NeoMTA 2 seems to be a promising material for vital pulp therapy. Further studies considering its biocompatibility and biomineralization potential are necessary.

## Introduction

The main objective of the dental pulp is to provide vitality to the tooth, since it is responsible for nourishing it by providing sensitivity, and responding to different stimuli such as pressure [1]. Certain dental injuries such as caries or trauma, if uncontrolled, could eventually lead to cellular death or necrosis. However, the pulp tissue, when affected in a reversible manner, presents an intrinsic reparative potential. Accordingly, the maintenance of pulp vitality by means of vital pulp therapy (VPT) procedures regained interest as a more conservative alternative to root canal treatment in cases of pulpitis [2]. Thus, this therapy can be defined as a restorative dental treatment and includes direct and indirect pulp capping, and pulpotomy, which can be partial or total [3].

Human dental pulp stem cells (hDPSCs) are considered a multipotential undifferentiated cell population with self-renewal capacity and the ability to repair dentin by dentin bridge formation [4]. These cells show a fibroblast-like morphology with superior proliferative ability compared to human bone marrow–derived mesenchymal stem cells. It has been shown that hDPSCs can differentiate into ectodermal-, mesodermal-, and endodermal-derived cells. Hence, these cells are being proposed as an alternative cell source for various reparative applications [5, 6].

Mineral trioxide aggregate (MTA) is considered the gold standard for vital pulp treatment due to its bioactivity, biocompatibility, hydrophilicity, and low solubility [7]. It is also reported to induce dental pulp proliferation, ion release, and hard tissue formation [8]. However, MTA has shown some disadvantages such as difficulty in handling, low compressive strength due to its porous matrix, and prolonged setting time that leads to washout in presence of excess moisture. To overcome the limitations in the clinical performance of MTA, new ion-releasing endodontic materials such as NeoMTA 2 (NuSmile Avalon Biomed, Bradenton, FL, USA) or Bio-C Repair (Angelus, Londrina, PR, Brazil) have been recently marketed [9].

Bio-C Repair is a new ready-for-use material with the same applications as MTA and has the ability to release calcium ions [10]. This new ion-releasing material contains zirconium oxide, calcium silicate, iron oxide, calcium aluminate, calcium oxide, silicon dioxide, and dispersing agent in its composition. In addition, previous reports have shown that this material exhibits similar biological effects and mineralization potential to MTA-based materials [9]. NeoMTA 2 is the second generation of root and pulp treatment materials whose predecessor was NeoMTA Plus (NuSmile Avalon Biomed, Bradenton, FL). Both materials are composed of a new tricalcium silicate–based material, with tantalum oxide (Ta_2_O_5_) as a radiopacifying agent instead of bismuth oxide to overcome its well-known discoloration potential [11, 12]. It is mixed with a water-based gel that results in superior handling properties. The mixing powder-to-liquid ratio can be varied depending on the indication for use: thin consistency as a sealer or thick consistency as a vital pulp material or perforation repair material [13]. Due to its recent introduction to the market, the effects of NeoMTA 2 on dental pulp cells are not fully understood in terms of biocompatibility or the expression profile of genes related to biomineralization.

A preliminary requirement for new materials is to establish the biological responses of cells exposed to them in vitro. In vitro tests are traditionally used as first-line testing to evaluate material properties including potential cytotoxicity [14].

Therefore, the objectives of this study were to analyze the biomineralization potential and biological effects of this new tantalum oxide (Ta_2_O_5_)–containing material (NeoMTA 2) and to compare these properties to those exhibited by NeoMTA Plus and Bio-C Repair. The null hypothesis of this study was that all ion-releasing endodontic materials exhibit a similar biomineralization potential and cytocompatibility.

## Materials and methods

### Tested materials and elution preparation

The tested materials in this study were as follows: NeoMTA Plus (NuSmile Avalon Biomed, Bradenton, FL, USA), NeoMTA 2 (NuSmile Avalon Biomed), and Bio-C Repair (Angelus, Londrina, PR, Brazil) (Table 1). Specimens of 2 mm diameter and 1 mm height were prepared and left undisturbed to set at 37 °C in 5% CO_2_ environment and 95% relative humidity for 48 h. Once set, the surfaces of the specimens were exposed for 20 min to ultraviolet light to ensure sterility. The grouping is depicted in Table 1. Each group was evaluated for cytotoxicity according to ISO 10,993–12 [15]. Final concentrations were 1/1, 1/2, and 1/4.

**Table 1:** The grouping of materials

Materials	Manufacturer	Composition	Composition
NeoMTA Plus	NuSmile Ltd (Avalon Biomed). 3315 West 12th Street Houston, TX 77,008 USA	**Powder**: Tricalcium silicate (Ca_3_SiO_5_), dicalcium silicate (Ca_2_SiO_4_), tantalum oxide (Ta_2_O_5_), and minor amounts of calcium sulfate (CaSO_4_) and tricalcium aluminate (Ca_3_Al_2_O_6_) **Liquid**: Water (H_2_O) and proprietary polymers	2,019,091,001
NeoMTA 2	NuSmile Ltd (Avalon Biomed). 3315 West 12th Street Houston, TX 77,008 USA	**Powder**: Tricalcium silicate (Ca_3_SiO_5_), dicalcium silicate (Ca_2_SiO_4_), tantalum oxide (Ta_2_O_5_), and minor amounts of calcium sulfate (CaSO_4_) and tricalcium aluminate (Ca_3_Al_2_O_6_) **Liquid**: Water (H_2_O) and proprietary polymers different from above	2,020,051,501
NeoMTA 2 has more tantalite and different polymers than NeoMTA Plus			
Bio-C Repair	Angelus, Rua Waldir Landgraf, Barrio Lindóia, Londrina, Brasil	Calcium silicate (Ca_3_SiO_5_), calcium aluminate (CaAl_2_O_4_), calcium oxide (CaO), zirconium oxide (ZrO_2_), iron oxide (Fe_2_O_3_), silicon dioxide (SiO_2_), and dispersing agent	531,522

### Ion release analysis

Test specimens with the aforementioned dimensions (*n* = 3) were prepared and stored at 37 °C in 100% humidity for 24 h. Each specimen was suspended in 5 mL deionized water for 1 day and the solution collected was analyzed using inductively coupled plasma-optical emission spectrometry (ICP-MS; Agilent 7900, Stockport, UK). The proportions of aluminum (Al), silicon (Si), sulfur (S), calcium (Ca), strontium (Sr), barium (Ba), and tungsten (W) released from each material were calibrated with pure deionized water. Analyzes were performed independently in triplicate (*n* = 3).

### Surface characterization

The surface and morphology analyses of the different materials were performed using SEM-coupled energy-dispersive spectroscopy (SEM–EDS, JSM-610LV, JEOL, Tokyo, Japan). Specimen disks of each material (*n* = 9) were prepared, and after setting time at 37 °C and 95% humidity, the disks underwent a carbon-coating process in a CC7650 SEM Carbon Coater unit (Quorum Technologies Ltd, East Sussex, UK). Finally, SEM micrographs were registered, and a qualitative analysis was performed for the surface element distribution.

### Isolation, culture, and characterization of human dental pulp stem cells

Cells were obtained from impacted third molars (*n* = 12) extracted for orthodontic reasons from patients aged 18–26 years old with prior informed consent using guidelines approved by the Institutional Committee of the University of Murcia (protocol ID: 2199/2018).

The extracted teeth were rinsed once in saline (0.9% w/v sodium chloride) and several times in sterile PBS, and then immersed in 1% povidone iodine for 2 min and 0.1% sodium thiosulfate in PBS for 1 min, and washed again in sterile PBS. Teeth were then vertically split to expose the dental pulp. The pulp tissue was gently extirpated and placed into an enzymatic bath containing collagenase type I (Gibco, Life Tech, NY, USA) at 37 °C for 40 min to digest the tissue and liberate the cells. Then, isolated cells were washed with PBS, filtered through 40-μm nylon cell strainers (BD Biosciences, San Jose, CA, USA), and cultured in DMEM supplemented with 10% fetal calf serum (Lonza, Basel, Switzerland), 1% GlutaMAX™ (Thermo Fisher Scientific, Waltham, MA, USA) and 1% penicillin/streptomycin (Thermo Fisher Scientific) (complete growth medium) at 37 °C and 5% CO_2_. Cell immunophenotype was performed by fluorescence-activated cell sorting analysis using specific antibodies for human CD14, CD20, CD34, CD45, CD73, CD90, and CD105, as described previously [15].

### Mitochondrial viability assay

hDPSC viability in presence of ion-releasing materials was performed by MTT (3-(4,5-dimethylthiazol-2-yl)-2,5-diphenyltetrazolium-bromide) assays. Briefly, 5 × 10^3^ cells per well were seeded in 96-well plates, allowed to attach for 24 h, and treated with several dilutions (1/1, 1/2, and 1/4) for 24, 48, and 72 h. Then, the medium was replaced with 5 mg/mL MTT under standard culture conditions for 4 h. In these assays, mitochondrial viability was measured by converting the MTT tetrazolium salt to a colored formazan compound by mitochondrial dehydrogenases. Finally, the absorbance was determined at 570 nm using a microplate reader (ELx800, Bio-Tek Instruments, Winooski, VT, USA) after cell lysis in 0.4 M hydrochloric acid solution in isopropanol (200 μl per well). Three independent experiments were performed for each sample and condition [15].

### Cell migration

The migratory ability of hDPSCs cultured with ion-releasing material eluates was determined using in vitro wound healing assays. hDPSCs were seeded into 12-well plates (2 × 10^4^ hDPSCs per well), and a vertical scratch was created using a 200-µL sterile-pipette tip. Microscopy images were then taken at 0, 24, 48, and 72 h and analysis was performed using ImageJ software (National Institutes of Health, Bethesda, MD, USA) [15]. Each experimental condition was carried out in triplicate for each material and analyzed in three independent experiments.

### Cell attachment

hDPSCs were cultured on the surface of the materials for 72 h. Afterwards, the culture medium was removed and cells were washed with PBS solution. Then, the adherent cells on the specimens were fixed with 3% glutaraldehyde for 30 min at 4 °C. The specimens were dehydrated using hexamethyldisilazane (Sigma-Aldrich, St. Louis, MO, USA) and varying concentrations of ethanol at room temperature. After fixation, the cell attachment was observed using scanning electron microscopy (SEM) after gold sputtering on the samples. Images were taken at × 100, × 300, and × 1500 magnifications [16]. Each experimental condition was carried out in triplicate for each material and analyzed in three independent experiments.

### Immunocytochemistry

Immunocytochemistry was performed to evaluate possible alterations in hDPSC morphology after exposure to undiluted ion-releasing materials. Briefly, hDPSCs were grown on coverslips for 72 h at 37 °C. Then, hDPSCs were fixed in 4% formaldehyde solution (Merck Millipore, Darmstadt, Germany) for 10 min and blocked with 5% bovine serum albumin (Sigma-Aldrich) for 30 min. The coverslips were stained with AlexaFluor™ 594–conjugated phalloidin (Invitrogen, Carlsbad, CA, USA) or phosphate-buffered saline in the control group. Afterwards, the nuclei were stained with 4,6-diamidino-2-phenylindole dihydrochloride (DAPI) (Thermo Fisher Scientific, Waltham, MA, USA). Finally, the coverslips were analyzed under a confocal microscope (Leica, Wetzlar, Germany) [16]. Each experimental condition was carried out in triplicate for each material and analyzed in three independent experiments.

### Apoptosis/necrosis assays and ROS production analyses

Cell viability and reactive oxygen species (ROS) production after exposure to the different material eluates were analyzed by annexin-V/7-AAD and the general oxidative stress indicator CM-H_2_DCFDA staining, respectively [17]. Briefly, hDPSCs were treated with complete culture medium alone (w/o any eluate) (control group) or with complete medium supplemented with different dilutions of the materials (1/1, 1/2, or 1/4) for 72 h at 37 °C. Afterwards, cells were washed and stained with FITC-conjugated annexin-V and 7-AAD (Immunostep, Salamanca, Spain) for 15 min at r/t, or with 5 μM CM-H_2_DCFDA (Molecular Probes, Eugene, OR, USA) for 30 min at 37 °C. Finally, samples were acquired in a BD LSRFortessa™ flow cytometer (Becton Dickinson, Franklin Lakes, NJ, USA) and percentages of live and apoptotic/necrotic cells or CM-H_2_DCFDA-positive cells were analyzed with FlowJo software (FlowJo LLC, Ashland, OR, USA). Each experimental condition was carried out in triplicate for each material and analyzed in three independent experiments.

### Gene expression analysis

The genetic expression of human alkaline phosphatase (*ALP*), collagen type 1 (*Col1A1*), dentin sialophosphoprotein (*DSPP*), osteonectin (*ON*), runt-related transcription factor 2 (*RUNX2*), and bone sialoprotein progenitor (*BSP*) was determined by quantitative polymerase chain reaction (qPCR) from cells treated with discs of the different material eluates after 14 days (*n* = 3) as described previously [16]. Briefly, cells were harvested and total RNA was isolated (Purelink RNA Mini Kit, Invitrogen, Thermo Fisher Scientific). Following that, cDNA synthesis was performed (iScript RT Supermix, Bio-Rad, Hercules, CA, USA). The 2^‑ΔΔCT^ method was used to calculate the relative gene expression values obtained by qPCR analysis for each gene compared to human glyceraldehyde 3-phosphate dehydrogenase (GAPDH) gene expression. Cells cultured with basal growth media were used as negative control, and cells treated with a commercial differentiation media (StemMACS OsteoDiff Media, Miltenyi Biotec, Bergisch Gladbach, Germany) acted as the positive control. Each experimental condition was carried out in triplicate for each sample and analyzed in three independent experiments.

### Alizarin Red S staining

To identify whether hPDSCs produced mineralized nodules in vitro in presence of NeoMTA Plus, NeoMTA 2, and Bio-C Repair eluates, Alizarin Red S staining was used according to previous studies [18]. Briefly, hPDSCs (2 × 10^4^ cells/well) were cultured in undiluted tested materials for 21 days. After the culture period, the culture medium was removed, and a 4% paraformaldehyde solution was added to each well for fixation for 15 min. The solution was removed, and the cells were washed with PBS for 10 min. Then, hPDSCs were stained with 2% Alizarin Red S solution (Sigma-Aldrich) (pH 4.1) for 15 min at r/t. The staining was solubilized with 10% cetylpyridinium chloride monohydrate (Sigma-Aldrich) solution and the absorbance of the eluted stains was read at 570 nm using a spectrophotometer. hDPSCs cultured in unconditioned medium (DMEM w/o any eluate) and OsteoDiff media were used as the negative and positive controls, respectively. Each experimental condition was carried out in triplicate for each material and analyzed in three independent experiments.

### Statistical analysis

All data represented the mean ± standard deviations (SD) of at least three independent culture experiments. Data were tested for normal distribution by the Kolmogorov–Smirnov test, and group comparisons were analyzed by one-way ANOVA, and differences between means were compared by Tukey’s multiple comparison test. The data obtained were analyzed using GraphPad Prism v8.0.2 (GraphPad Software, San Diego, CA, USA). *p*-values < 0.05 were considered statistically significant.

## Results

### Ion release

The results from ion release analysis are shown in Table 2. In general terms, NeoMTA 2 exhibited a higher release of Ca^2+^ (23.03 ± 0.02) compared to NeoMTA Plus (9.04 ± 0.01) and Bio-C Repair (15.01 ± 0.00) (*p* < 0.05), while strontium (Sr) and silicon (Si) ion release were significantly increased in Bio-C Repair (*p* < 0.05). In contrast, NeoMTA Plus was associated with the highest release of sulfur (S) and tungsten (W).

**Table 2:** Assessment of ICP-MS of ion-releasing materials

	29 Si [He]	34 S [He]	42 Ca [He]	88 Sr [He]	137 Ba [He]	181 Ta [He]	182 W [He]
Sample name	Conc. [ppm]	Conc. [ppm]	Conc. [ppm]	Conc. [ppm]	Conc. [ppm]	Conc. [ppm]	Conc. [ppm]
NeoMTA Plus	6.75 ± 0.00^AB^	7.80 ± 0.01^AB^	9.04 ± 0.01^AB^	425.48 ± 0.01^AB^	5.23 ± 0.04^A^	0.12 ± 0.02^AB^	59.66 ± 0.00^AB^
NeoMTA 2	2.40 ± 0.02^AC^	1.79 ± 0.01^AC^	23.03 ± 0.02^AC^	212 ± 0.01^AC^	3.57 ± 0.03^C^	0.48 ± 0.02^BC^	1.86 ± 0.00^AC^
Bio-C Repair	19.57 ± 0.00^BC^	3.64 ± 0.02^BC^	15.01 ± 0.00^BC^	928 ± 0.03^BC^	10.77 ± 0.00^AC^	< 0.000 ± 0.000^AC^	8.52 ± 0.00^BC^

Uppercase A indicates significant difference (*p* < 0.05) between NeoMTA Plus and Bio-C Repair

Uppercase B indicates significant difference (*p* < 0.05) between NeoMTA Plus and NeoMTA 2

Uppercase C indicates significant difference (*p* < 0.05) between NeoMTA 2 and Bio-C Repair

### SEM–EDS analysis

**Fig. 1:**
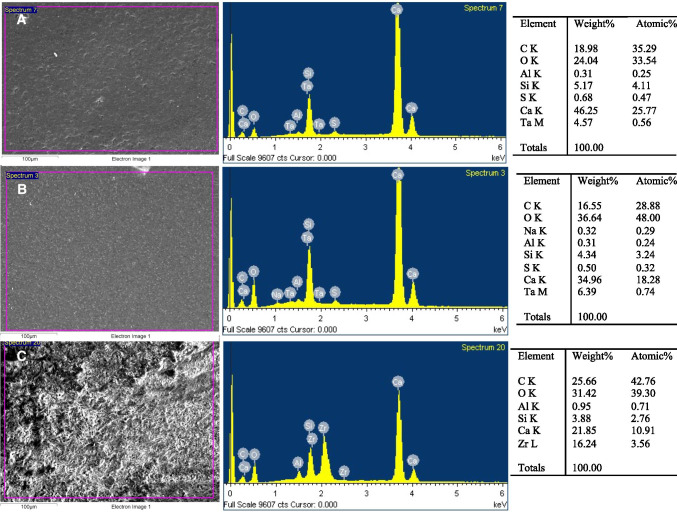
SEM–EDS analysis results for NeoMTA 2 (**A**), NeoMTA Plus (**B**), and Bio-C Repair (**C**) disks (*n* = 9). The first column presents SEM micrographs of each material (scale bar: 100 μm). The second column illustrates the EDS plots with the correspondent peaks detected. The third column classifies the list of elements present per material by weight and atomic weight

The EDX analysis of the chemical elements on the surface of the specimens was comparable to the material composition according to the manufacturers’ information, as shown in Fig. 1 and Table 1. EDX analysis of NeoMTA 2 displayed a higher peak of Ca^2+^ than NeoMTA Plus and Bio-C Repair. Peaks of Zr^+^ and Si^4+^ were also observed in Bio-C Repair due to the presence of ZrO_2_ and silica, while tantalum (Ta^5+^) was detected in NeoMTA 2 and NeoMTA Plus. Additionally, the SEM–EDS analysis disclosed other elements which were not mentioned in the manufacturers’ information: aluminum (Al^3+^) and sulfur (S^2−^) were found in NeoMTA 2 and NeoMTA Plus, while aluminum (Al^3+^) alone was detected in Bio-C Repair.

### Mitochondrial viability assay

**Fig. 2:**
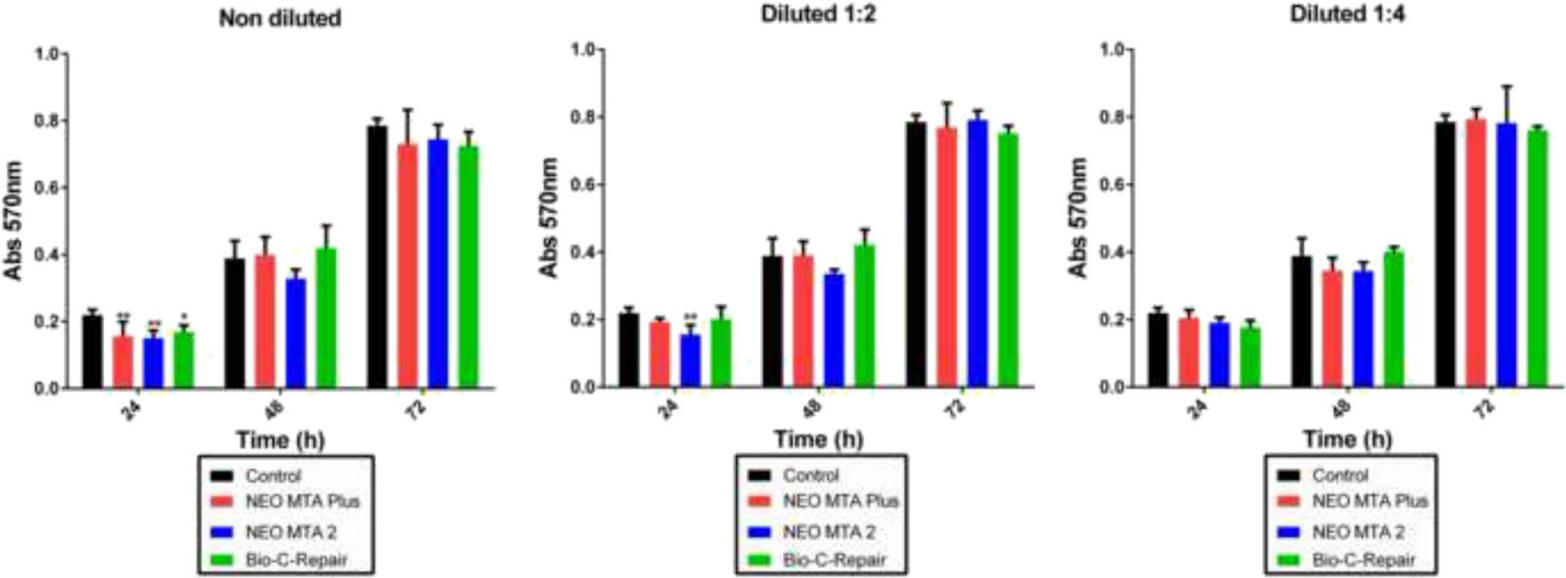
Mitochondrial viability assay. In vitro cytotoxicity of hDPSCs after exposure to extracted medium prepared from Neo MTA Plus, Neo MTA 2, and Bio-C Repair. Data are presented as absorbance values (570 nm) at 24, 48, and 72 h of exposure of the material eluates to hDPSCs, compared to the control. *p < 0.05; **p < 0.01; ***p < 0.001. Each experimental condition was performed in triplicate for each VPT material and analyzed in three independent experiments

After 72 h of culture, the exposure of hDPSCs cells to the material eluates did not significantly affect mitochondrial metabolism (Fig. 2). Only with undiluted materials, a significant reduction in cell viability was evidenced, compared to the negative control in the first time point (24 h) (*p* < 0.05, *p* < 0.01).

### Migration assay

**Fig. 3:**
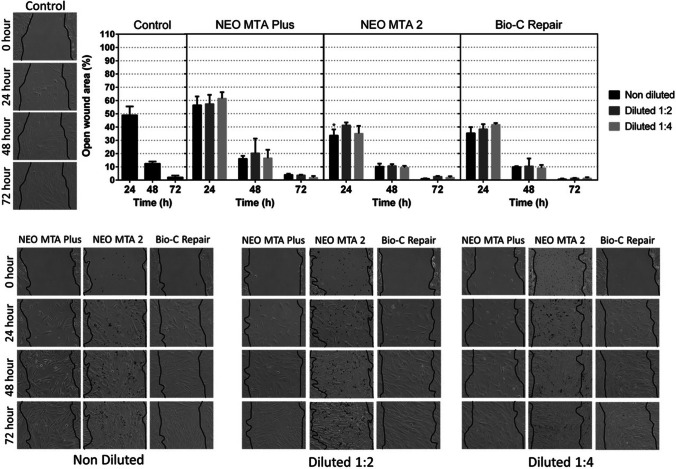
Migration was evaluated using wound healing assays. Cells were exposed to undiluted (1/1) and diluted (1/2 and 1/4) eluates from materials. The control condition consisted of cells maintained in normal growth medium. Graphical results are presented as mean relative wound closure (RWC) percentages at each of the time points, relative to the total wound area at 0 h. Asterisks designate significant differences compared to the control. *p < 0.05; **p < 0.01; ***p < 0.001. Each experimental condition was performed in triplicate for each VPT material and analyzed in three independent experiments

Cell migration ability was monitored by wound healing assays. hDPSCs cultured in basal growth medium served as the negative control. No significant differences were found between any of the ion-releasing materials and the negative control at 24 and 48 h (Fig. 3). Only at 24 h, undiluted NeoMTA 2 promoted a higher cell migration than the control group (*p* < 0.05). These results confirmed that all materials allowed an adequate cell migration.

### Cell attachment

**Fig. 4:**
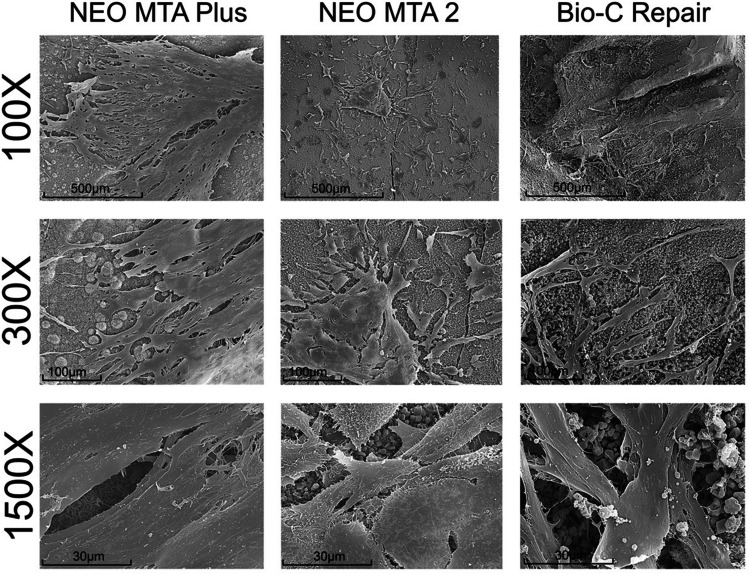
Cell attachment. Sample disks with the aforementioned standardized dimensions were obtained (*n* = 15) for each of the materials and allocated into three groups (*n* = 5). Representative SEM micrographs illustrate the adhesion of hDPSCs directly seeded on Neo MTA Plus, Neo MTA 2, and Bio-C Repair. Magnifications: × 100, × 300, and × 1500. Scale bars: 500 μm, 100 μm, and 30 μm

The cellular attachment and morphology can be affected by exposure to cytotoxic agents and directly reflects cell injuries. The analysis of cell attachment of hDPSCs on the surfaces of the different ion-releasing materials was observed after 3 days of culture. Abundant well-adhered and functionally oriented cells were observed in all materials, suggesting no cytotoxic effect (Fig. 4).

### Cell cytoskeleton staining

**Fig. 5:**
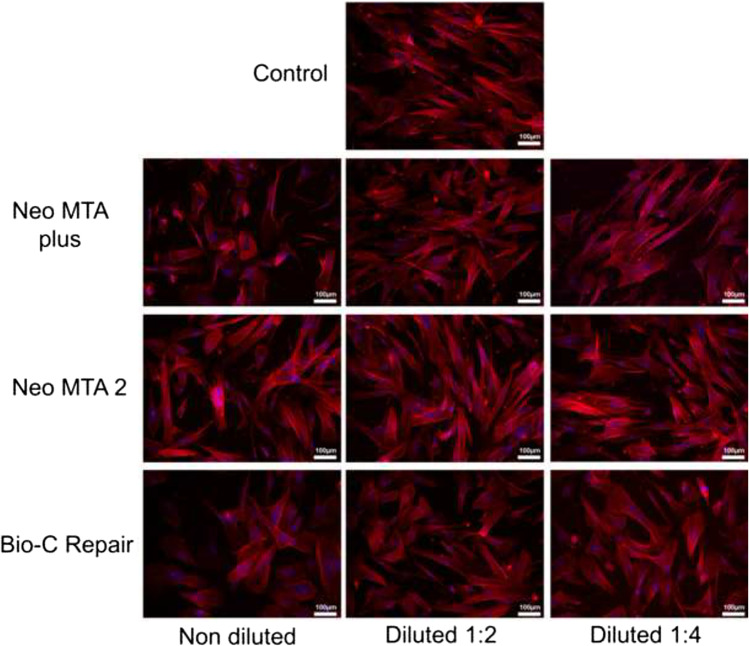
Analysis of changes in cell morphology, actin cytoskeleton structure, and organization on hDPSCs after treatment with NeoMTA Plus, NeoMTA 2, and Bio-C Repair by confocal fluorescence microscopy. F-actin fibers were stained with AlexaFluor™ 594–conjugated phalloidin (red), whereas cell nuclei were counterstained with DAPI (blue). Confocal fluorescence microscopy images shown are representative from *n* = 3 separate experiments. Scale bar: 100 μm. Each experimental condition was performed in triplicate for each VPT material and analyzed in three independent experiments

All ion-releasing materials exhibited positive phalloidin staining with a fibroblastic spindle-shaped morphology and increased F-actin content, indicating the contractile property of hDPSCs. A similar phenomenon was observed on the cells from the negative control group (Fig. 5).

### Apoptosis/necrosis and ROS production assays

**Fig. 6:**
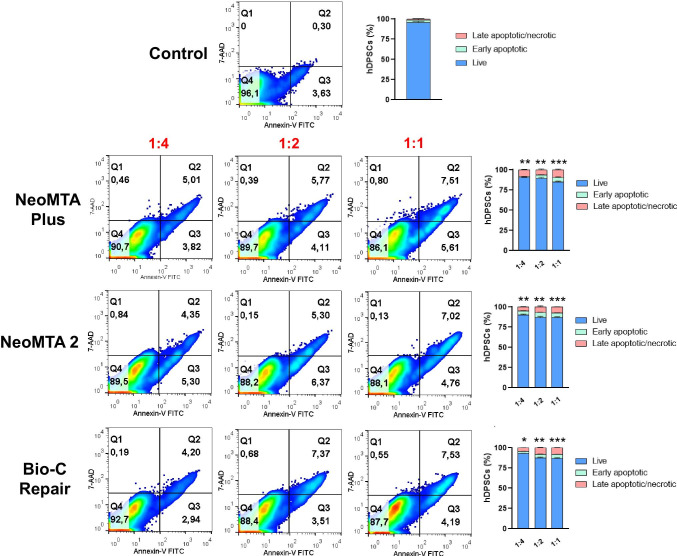
Flow cytometry analysis of cell apoptosis and necrosis induced by the different vital pulp material extracts on hDPSCs by annexin V-PE/7-AAD staining. Numbers inside representative dot plots represent percentages of live (Q4 quadrants), early apoptotic (Q3 quadrants), and late apoptotic and necrotic cells (Q1 and Q2 quadrants). Bar graphs show mean ± SD from *n* = 3 separate experiments. Percentages of live cells were significantly decreased compared to the control, *p < 0.05; **p < 0.01; ***p < 0.001, respectively

**Fig. 7:**
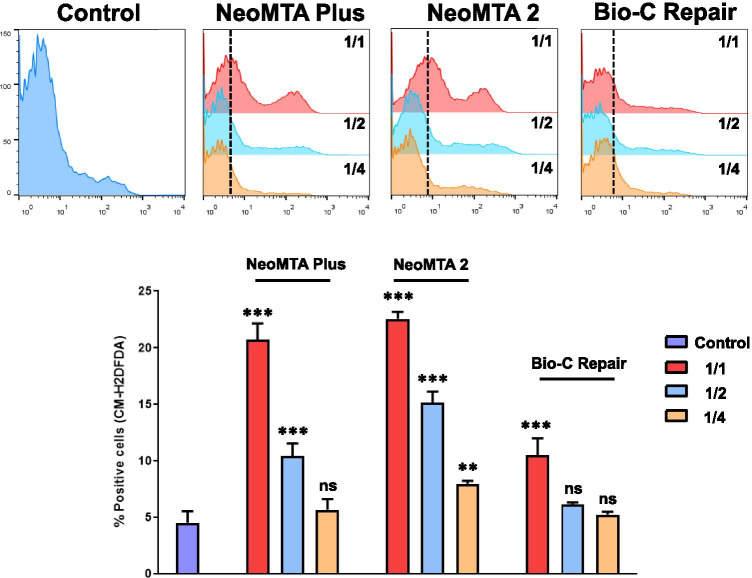
Reactive oxygen species (ROS) production. CM-H_2_DCFDA staining was used to evaluate intracellular ROS production in hDPSCs exposed to ion-releasing materials. Bar graph showing percentages of positive cells for ROS production in each experimental condition is shown and represented as mean ± SD from *n* = 3 separate experiments. Percentages of CM-H_2_DCFDA-positive cells were significantly increased compared to the control, *p < 0.05; **p < 0.01; ***p < 0.001, respectively

The cell viability after exposition to several dilutions of each material (1/1, 1/2, and 1/4) was analyzed by annexin-V/7-AAD staining by flow cytometry. After 72 h of culture, hDPSCs in presence of 1/4 dilutions of each material did not show significant levels of apoptosis or necrosis compared to control cells. Only undiluted extracts (1/1) caused a slight decrease in cell viability (⁓ 10%) (Fig. 6). Moreover, as shown in Fig. 7, percentages of CM-H_2_DCFDA-positive cells were progressively decreasing from 1/1 to 1/4 dilutions of each material compared to control cells, especially for tantalum oxide (Ta_2_O_5_)–containing materials (NeoMTA Plus and NeoMTA 2). Among them, CM-H_2_DCFDA-positive cells after NeoMTA 2 treatment were significantly increased at every dilution compared to the same dilutions of the other materials and control cells.

### RT-qPCR assay

**Fig. 8:**
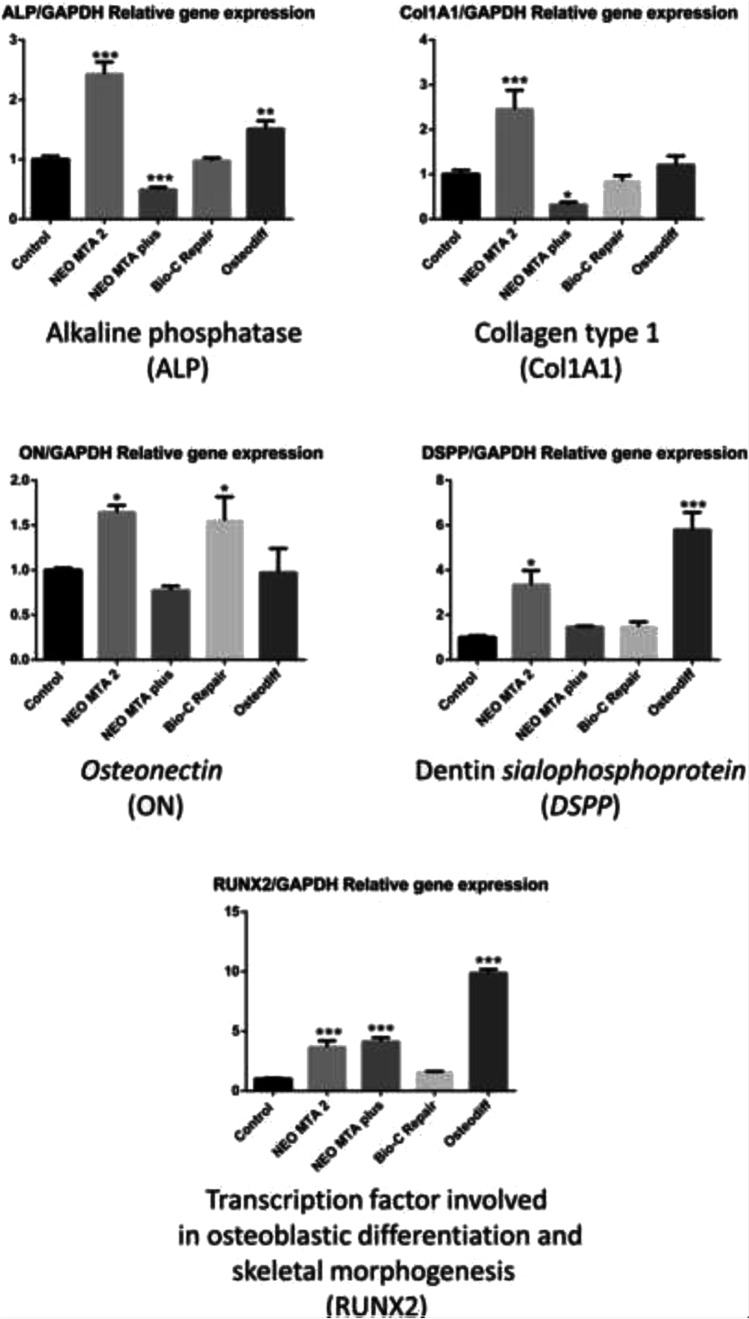
The expression of osteo/odontogenic genes detected by RT-qPCR. Data are expressed as mean ± SD and relative to *GAPDH* gene expression. *p < 0.05; **p < 0.01; ***p < 0.001. Each experimental condition was performed in triplicate for each VPT material and analyzed in three independent experiments

To evaluate the effect of ion-releasing materials in promoting odontogenic gene expression of hDPSCs, cells were cultured up to 14 days with either control negative medium, osteogenic inducing medium (Osteodiff), or medium containing ion-releasing materials. As shown in Fig. 8, hDPSCs treated with NeoMTA 2 displayed an upregulation of *ALP*, *Col1A1*, *RUNX2* (*p* < 0.001), *ON*, and *DSPP* genes (*p* < 0.05) compared to the negative control, while Bio-C Repair and NeoMTA Plus groups showed only upregulation of *ON* (*p* < 0.05) and *RUNX2* (*p* < 0.001) respectively. Finally, when hDPSCs were cultured in an osteogenic inducing medium (Osteodiff group), upregulated expression of *DSPP*, *RUNX2* (*p* < 0.001), and *ALP* (*p* < 0.01) was observed when compared to the negative control at day 14.

### Mineralization assay

**Fig. 9:**
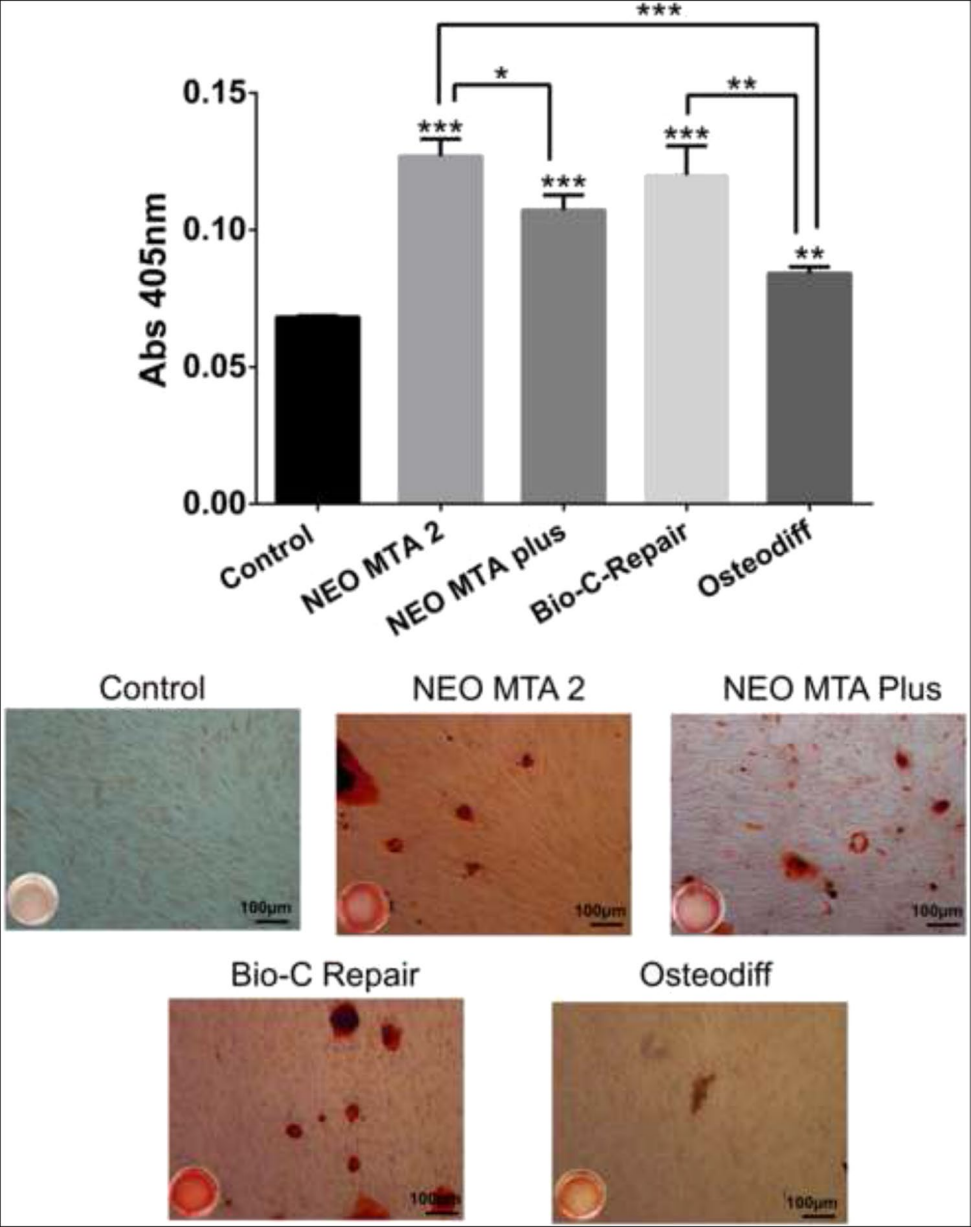
Calcium deposition, as a final product of the odonto/osteogenic differentiation process, was assessed by using Alizarin Red S staining on day 21 (*p < 0.05; **p < 0.01; ***p < 0.001). Each experimental condition was performed in triplicate for each VPT material and analyzed in three independent experiments

The potential for calcium deposition of ion-releasing materials was identified by using Alizarin Red S staining on day 21 (Fig. 9). All hDPSCs treated with the undiluted materials and in the osteogenic induction medium exhibited deposition of calcium nodules. There were significant differences between the tested groups, with NeoMTA 2 showing the highest mineralization potential (*p* < 0.001). In contrast, the control group containing hDPSC cells cultured in the basal culture medium without any materials did not show mineralized nodule deposition.

## Discussion

The purpose of this study was to analyze the cytocompatibility and biomineralization ability of the three ion-releasing materials. In vital pulp therapy, materials play an important role in the proliferation, differentiation, and calcium nodule deposition of hDPSCs [19]. Moreover, successful vital pulp therapy also depends on the placement of a bioactive and biocompatible material which promotes human tooth-pulp cells to create a reparative dentin barrier to protect the pulp tissue from external noxious agents [7, 20, 21]. For this reason, human dental pulp stem cells were used as the target cells for the laboratory tests. Furthermore, immortalized cells are genetically modified and may exhibit clinically inappropriate toxic responses to compounds [22].

Some studies on cytotoxicity of ion-releasing materials have been previously reported in human cells, for example, apical papilla [23] and endothelial cells [24], while others have been tested on animal cells [25]. However, to our best knowledge, the potentially toxic effects of NeoMTA on human stem cells remain unknown. In addition, the evaluation of these materials was performed via incubation of the cultured cells with several dilutions of the materials (1:1, 1:2, and 1:4). This was done to simulate the clinical conditions in which the materials will be applied, since they are placed on remaining dentin thicknesses of 0.01 to 0.25 mm or in cavities with pulp exposure and the concentration of material which reaches the viable tissue may vary [23, 26].

Regarding ion release, NeoMTA 2 was associated with the higher release of calcium ions from all of the tested materials (*p* < 0.05). It has been described that Ca^2+^ release stimulates hydroxyl apatite formation and release of alkaline phosphatase and bone morphogenetic protein 2, which are important in the mineralization process [27]. Both SEM–EDS and ICP-MS assays exhibited Ca^2+^ release and calcium content in all materials, as previously described for NeoMTA Plus and Bio C-Repair [28, 29]. Moreover, tantalum (Ta^5+^) was detected both in NeoMTA 2 and NeoMTA Plus. A recent report described that the incorporation of Ta^5+^ as an alternative radiopacifier does not cause discoloration and preserves acceptable values of radiopacity [30]. Also, Si^4+^ release was detected in all ion-releasing materials after setting (*p* < 0.05). Other than the release of Ca^2+^ ions, other ions, namely silicon dioxide, aluminosilicates, and CaO, are also released during hydration and could have also enhanced the cellular differentiation and proliferation [31]. The differences in ion release may also influence the role of calcium silicates in upregulating the expression of genes related to mineralization by hDPSCs [32].

The mitochondrial viability assay revealed that after 24 h, undiluted materials and 1:2 NeoMTA 2 slightly affected the mitochondrial metabolism of hDPSCs. This finding may be related to the initial dissociation of Ca^2+^ during the first 24 h. In fact, excessive intracellular accumulation of Ca^2+^ may lead to mitochondrial dysfunction, alteration of cytoskeleton organization, and activation of catabolic enzymes [33]. However, both the MTT assay results and flow cytometry results after annexin-V/7-AAD staining showed that hDPSCs were not affected by any of the tested material concentrations after 48 h and 72 h of culture, compared to the control (*p* > 0.05). Besides, significant oxidative effects were not evidenced, especially with NeoMTA 2 and NeoMTA Plus. These results suggest that these materials did not exhibit an apparent cytotoxic effect after 48 h and 72 h. These findings are consistent with previous reports showing that NeoMTA Plus and Bio-C Repair elutes were not cytotoxic [28, 34].

Cell migration is a crucial factor in angiogenesis, and it has been described that released substances could potentially delay or enhance the angiogenesis and healing process [35]. For this reason, scratch assays were performed in order to preliminarily predict how the coordinated migration of hDPSC would occur during pulp inflammation or after injury. In agreement with our observations on the accumulation of MTT formazan assay, no important differences were apparent between the groups, exhibiting an optimal migration speed with these tested ion-releasing materials. In fact, cell migration rates were the highest when treated with the highest dilution (1:4) of NeoMTA 2 at 24 h (*p* < 0.05). A similar tendency for cell migration was observed by Mestieri et al*.* who also found an increased cell migration with ion-releasing materials, corroborating our findings [36]. The cell migration results correlated with the observed cell adhesion, explained by the fact that cellular morphology can be affected by exposure to cytotoxic agents and directly reflects cell injuries [37]. Furthermore, changes in cellular morphology have been considered a direct indicator in assessing cytotoxicity [38]. In this study, hDPSCs adopted a spread morphology and adequate attachment when exposed to ion-releasing materials. Hence, these results suggest that the tested materials did not affect cell attachment and migration.

Our results showed that a new tantalum oxide (Ta_2_O_5_)–containing material (NeoMTA 2) increased the expression of osteo/odontogenic-related genes after 14 days compared to the untreated control. ALP is an enzyme which is present in the osteoblast membrane and participates in bone matrix synthesis and mineralization [39]. The increase in this enzyme’s activity is considered an early marker of stem cell differentiation in pre-osteoblasts and osteoblasts [40]. The early expression of osteogenic-related markers seems to be consistent across studies when pulp cells are treated with calcium silicate–based cement [4, 41]. Previously, high gene expression of osteocalcin (OCN) was observed in human dental pulp cells exposed to BioAggregate, iRoot BP Plus, and MTA for 7 days [42] and positive expression of osteopontin (OPN) protein was observed at an early time point in the dental pulp of dogs treated with MTA and Biodentine [43]. The differences in the expression profiles may be explained by the fact that OCN and OPN markers are expressed in late polarizing odontoblasts and secretory odontoblasts while DSPP and DMP-1 expression is higher in terminally differentiated and secretory or functional odontoblasts [44]. However, one limitation of this study is the lack of direct comparability of its results due to the absence the studies on the differentiation potential of NeoMTA 2.

The capacity for calcium deposition has been identified as an indicator of successful odontoblastic differentiation [45, 46]. hDPSCs showed deposition of calcium nodules after 21 days of culture in the presence of ion-releasing materials and in the osteogenic induction medium, as indicated by the staining with Alizarin Red S. Also, the control groups containing hDPSCs cultured in the basal medium and without extracts did not present mineralized nodules. It has been reported that the presence of tantalum and zirconium promotes the osteo/odontogenic differentiation of hDPSCs [4]. Previous studies showed that iRootBP Plus and NeoMTA Plus, which contain Ta_2_O_5_, promote mineralization and the formation of reparative dentine bridges in vitro and in vivo [47, 48]. The higher mineralization potential exhibited by NeoMTA 2 than his predecessor NeoMTA Plus may be related to the higher proportion of tantalite and different polymers. Again, no available evidence on mineralization potential was found regarding NeoMTA 2. However, future laboratory studies are necessary to understand the exact mechanisms of the induction of osteogenic differentiation and deposition of mineralized matrix stimulated by new ion-releasing materials. Also, physicochemical properties as radiopacity, flowability, or setting time should be considered in future studies.

## Conclusions

Within the limitations of this in vitro study, it can be concluded that NeoMTA 2 promotes cell viability, cell migration, and cell attachment, and induces the odonto/osteogenic differentiation of hDPSCs without using chemical osteogenic inducers.
